# Supplementary figures and images for: Estimating household contact matrices structure from easily collectable metadata
Source: PLoS One. 2024 Mar 14;19(3):e0296810. doi: 10.1371/journal.pone.0296810 (PMC10939291; doi:10.1371/journal.pone.0296810)

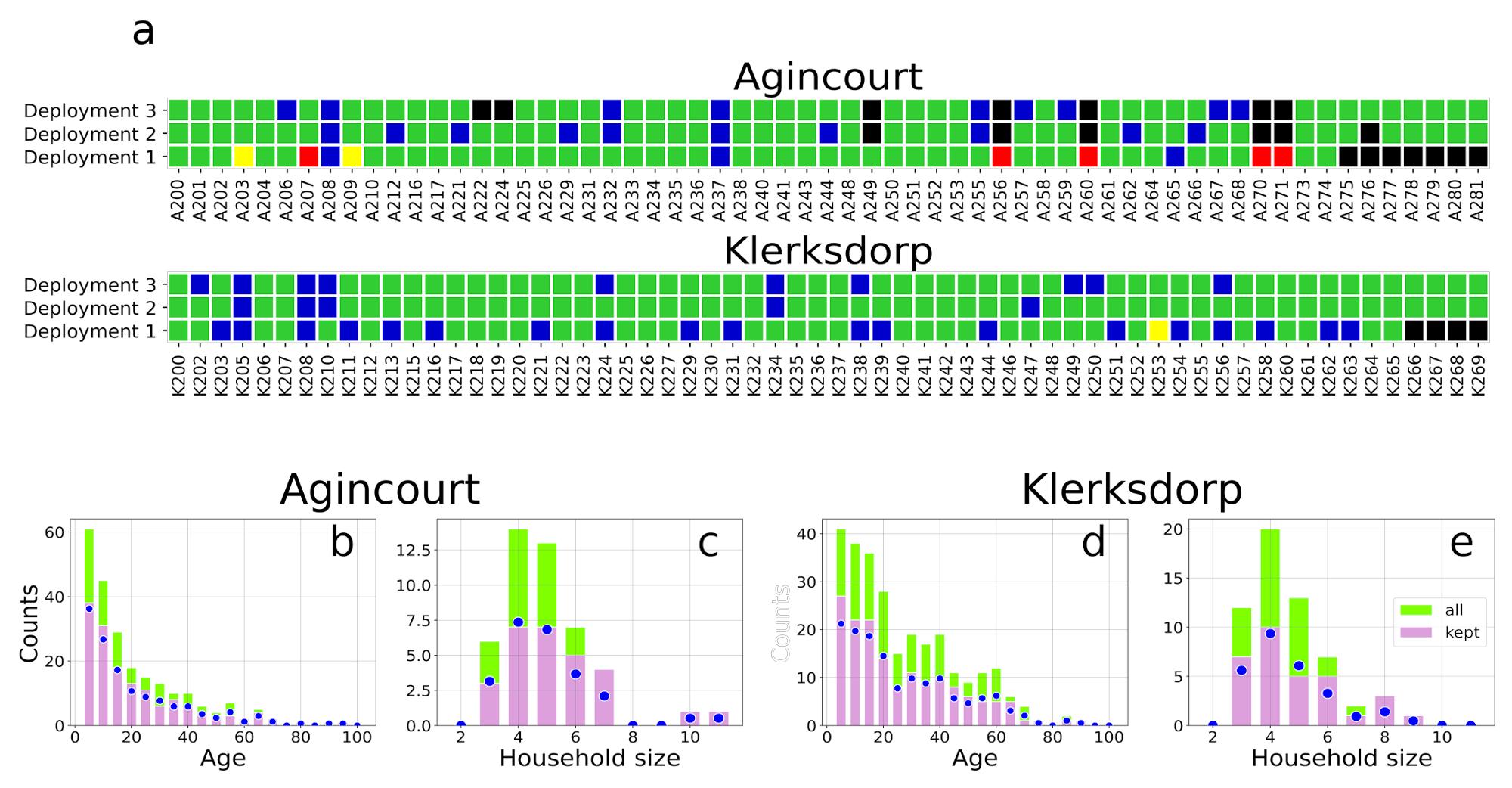

Supplement: S1 Fig — a: data quality. On the x-axis we plot households, while on the y-axis the deployments. For each (household-deployment) we assign a color code: black indicates that the household did not participate; red that all household’s sensors had data quality issues and did not provide valid measurements; blue that there are less than two days of measurement; yellow that a non circadian activity is observed; green none of the above. b and d: age distribution in Agincourt and Klerksdorp, respectively. The green bars are referred to the whole data-set, while the purple one only refers to the 60 households with valid measurements in all three deployments (see a). Blue dots are obtained by multiplying the height of the green bars for the fraction of the included households, that is the expected bar height, given the cleaned dataset size. c and e: household size distribution. Legends and colors follow the description of b and d. (TIFF) [file pone.0296810.s001.tiff]
