# Supplementary material for: Estimating household contact matrices structure from easily collectable metadata
Source: PLoS One. 2024 Mar 14;19(3):e0296810. doi: 10.1371/journal.pone.0296810 (PMC10939291; doi:10.1371/journal.pone.0296810)
Supplement: S1 Appendix — (PDF) [file pone.0296810.s002.pdf]

S Supplementary information: Appendix

S.1 Data collection and pre-processing

Proximity data are measured with the `SocioPatterns` sensors that we here introduce, addressing the interested reader to [1] for a more detailed reference. Their functioning is based on the emission of low-power signals. Participants are asked to wear the sensor on their chest, so that when they engage in a face-to-face interaction with another participant, the respective sensors can exchange packets of information with a frequency that does not exceed one packet per second. A contact is measured if, in the time-span of 20 seconds, two sensors exchange at least one packet, recording the unique identifier of the interacting sensor, the time at which the interaction occurred and the attenuation of the signal from the sender to the receiver. This attenuation is related to the distance between the two sensors and can be used to filter suitably-defined close-range proximity relations. Additionally, each sensor periodically records some status properties that log metadata and diagnostic information. Among these, an accelerometer allows one to know every 15 minutes if the sensor is moving or not. Given the sensitivity of the accelerometer and the time-scale at which it operates, one can assume that if the sensor is still, then it is not worn. The cleaning procedure is summarized as follows:

1. All contacts measured by non-moving sensors are removed: this is to avoid including spurious contacts between sensors that are, for instance, kept inside a drawer

2. Contacts are filtered and only those with a suitable attenuation threshold. This threshold corresponds to an interaction between two sensors that are approximately at 2 meters, even if this is a context-dependent relation that depends on external parameters, such as, for instance, humidity.

3. All contacts happening before the beginning of the deployment (as reported in the diaries) and after its end are removed. These contacts may exist, because sensors may be collected on different dates from the ones of the planned experiment, but they are removed because sensors' use may be non-systematic, hence unreliable. Moreover, the first and last day of measurement are removed as well. During these days, very intense activity patterns are typically observed due to the interaction with the people dispatching the sensors. Since this kind of interaction deviates from the standard conditions, it is not considered.

4. The data collected by the sensors contains information on the hardware identification code. A mapping relates this identifier with the individuals' pseudonym that allows us to relate contacts and metadata. Errors at this stage make it impossible to relate contacts to people and results in the red dots shown in Figure S1a.

5. As a minimal request, we impose that, after this cleaning procedure, a deployment can be considered valid only if it has two or more days of measurement. We found this to be a good trade-off between high quality data to work with and a sufficiently comprehensive inclusion principle. Household-deployment pairs that do not fulfill this condition are denoted in blue in Fig S1a.

6. Finally, non-circadian activity patterns are identified. A great excess of activity during night hours was observed in three households (yellow dots of Figure S1a) during the first deployment. This may occur, for instance, if the sensors are left in proximity on a vibrating surface: the accelerometer filter does not remove these contacts even though the sensors were not worn at that moment.

Only the households in which all three deployments led to valid measurements (all green dots in Fig S1a) were included in our study. Figure S1b, c, d, e further show the age and household size histograms for the whole dataset against its cleaned version, showing that our inclusion principle did not affect either of the four distributions.

S.2 Validation approach

Sampling the villages

First of all, in order to devise a good approximation of HCM, it is necessary to define a suitable distance to compare them. When comparing different households, however, one has to consider that typically there are some age groups with no individuals. More formally, this means that for some age group a, $\Phi_a = 0$. In some extreme cases there is no way to consistently compare HCM because the corresponding contact matrices are complementary, *i.e.* the zeros one correspond to the non-zeros of the other.

To address this problem, we choose to compare groups of HCM (*villages*) $\mathcal{X}$, *i.e.* small groups of household-deployment (h, d) pairs that guarantee that $\Phi_a > 0$ for all a. To build the samples $\mathcal{X}$ we then first select some (h, d) at random, with the constraint to achieve $\Phi_a > 0$ for all a (we sample only the pairs that can contribute to increasing the zeros entries of this vector) and then we randomly pick other pairs until the fixed size of $\mathcal{X}$ is reached. We empirically choose $|\mathcal{X}| = 8$ because it is a good trade-off between two competing effects: if $|\mathcal{X}|$ is too low there is a possibility of over-representing households with elderly members that are fewer and hence more valuable to get the condition $\Phi_a > 0$ for all a; on the other hand, very large values of $|\mathcal{X}|$ will tend towards an "averaging" effect that leads all *villages* to be very similar to one-another.

Model calibration

Given the samples of villages we now compute the value $\boldsymbol{u}$ as the result of the following optimization problem

$$\boldsymbol{u} = \underset{\boldsymbol{v}\,:\,\boldsymbol{v}^T\mathbf{1}=\text{const}}{\arg\min}\; d_{\text{C}}\left(C, T \circ \boldsymbol{v}\boldsymbol{v}^T\right),$$

where $\left[T \circ (\boldsymbol{v}\boldsymbol{v}^T)\right]_{ab} = T_{ab} v_a v_b$ and d_{C} is a modified Canberra distance. Let A, B be two symmetric matrices of size n_{age}, then

$$d_{\text{C}}(A, B) = \sum_{i=1}^{n_{\text{age}}} \sum_{j \le i} \frac{|\tilde{A}_{ij} - \tilde{B}_{ij}|}{|\tilde{A}_{ij}| + |\tilde{B}_{ij}|}$$

where $\tilde{A}$ is the matrix A divided by its mean (and equivalently $\tilde{B}$ is B divided by its mean). The distance d_{C} is the Canberra distance computed on the matrices $\tilde{A}, \tilde{B}$, instead of A, B, hence we refer to it as *modified Canberra distance.* This choice of the distance is motivated by the two following points

1. The entries of C may differ even by a factor 100 as shown in Figure **??**. The cosine similarity is meaningful to quantify the proximity of two matrices but it naturally tends to give more weight to entries with a larger magnitude. For this reason it is unsuited for an optimization as it would poorly estimate the small entries of C. On the opposite, the relative distance d_{C} gives approximately the same weight to all matrix entries and can be used for this purpose.

2. The modified Canberra distance compares a normalized version of the contact matrices because we are interested in determining them up to a constant factor. We then have for any $\alpha, \beta > 0$, $d_{\mathrm{C}}(\alpha A, \beta B) = d_{\mathrm{C}}(A, B)$.

Occupation parameter

We here detail the strategy to determine the vectors $\boldsymbol{y}, \boldsymbol{\eta}$ appearing in Figure **??**b, c, referred to as *occupation* and *compliance* vector respectively.

In the PHIRST data collection process, the participants were asked to specify locations or activities in which they spend more than three hours a day for more than three days per week. The options to choose from included: school, university, work, pub, social clubs, hanging out with friends, street vendors and church. We then define a Boolean variable for each person indicating whether or not he/she has a major activity outside the household, *i.e.* if he/she answered positively to *any* of the questions above. The value of y_a is the average of the Boolean indicator for all people of age a in $\mathcal{X}$.

References

1. Cattuto C, Van den Broeck W, Barrat A, Colizza V, Pinton JF, Vespignani A. Dynamics of person-to-person interactions from distributed RFID sensor networks. PloS one. 2010;5(7):e11596.
